# Ethylenediaminetetraacetic acid (EDTA) alters phytochemicals of cooked *Vicia faba* beans and impairs liver and kidney functions in CD-1 mice

**DOI:** 10.1038/s41598-026-61448-z

**Published:** 2026-08-10

**Authors:** Sabry A. El-Naggar, Mona M. Elwan, Soha I. Sakr, Hany M. El-Wahsh, Karim S. El-Sadi, Doaa I. Kabil, Mohamed A. Kelany, Kadry M. Sadek

**Affiliations:** 1 Zoology Department, Faculty of Science, Tanta University, Tanta, 31527 Egypt; 2 Home Economics Department, Faculty of Specific Education, Tanta University, Tanta, Egypt; 3 Faculty of Marine Sciences, Marine Biology Department, King Abdulaziz University, Jeddah, Saudi Arabia; 4 Biochemistry Division, Chemistry Department, Faculty of Science, Tanta University, Tanta, 31527 Egypt; 5 Department of Food Science and Nutrition, Faculty of Agriculture, Sohag University, Sohag, 82524 Egypt; 6 Department of Biochemistry, Faculty of VeterinaryMedicine, Damanhour University, Damanhour, Egypt

**Keywords:** EDTA, GC-MS, Sub-acute, Sub-chronic-toxicity, Hepatorenal pathology, Phytochemicals, *Vicia faba* beans, Biochemistry, Biological techniques, Chemical biology, Drug discovery, Plant sciences

## Abstract

*Vicia faba* beans are important food source of plant-based protein. In culinary practices, various chemicals are sometimes added to accelerate the cooking of *V. faba*. This study aimed to evaluate the effects of ethylenediaminetetraacetic acid (EDTA) addition during the cooking process of *V. faba* beans on their phytochemical compositions in addition to addressing the potential hepato-renal toxicity associated with short- and long-term administration of the EDTA-containing cooking medium (CM/EDTA).Phytochemical components of both raw and cooked *V. faba* beans were analyzed using the biochemical methods and gas-chromatography mass spectrophotometry (GC-MS). Ninety CD-1 male mice were randomly divided into six groups (*n* = 15). Groups 1 and 2 (Gp1 and Gp2) served as negative controls. Groups 3 and 4 (Gp3 and Gp4) received oral administration of CM or CM/EDTA (10 mg/kg) for 15 days (Sub-acute), while groups 5 and 6 (Gp5 and Gp6) received the same doses as in Gp3 and Gp4 for 90 days (Sub-chronic). Biochemical, molecular, and histopathological assessments were performed to evaluate liver and kidney functions. The results revealed that the addition of EDTA during cooking process led to marked alterations in the phytochemical compositions of *V. faba* beans evidenced by the biochemical and GC-MS analysis. Results showed that the ethyl α-D-glucopyranoside was reported in seeds extract (SE), while this compound disappeared in SE after cooking (C) and SE/C/EDTA. Only, SE/C/EDTA contains di-isooctyl phthalate (DIOP). Sub-chronic administration of CM/EDTA resulted in significant biochemical, molecular, and histopathological changes in hepatic and renal tissues of mice, indicating potential toxicity associated with prolonged exposure.

## Introduction

One edible legume crop used for human nutrition is the faba bean (*Vicia faba* L.). In Asia, Africa, and South America, faba beans constitute a staple food^1^. It belongs to the Leguminosae family of Fabaceae. It is believed to extend the shelf life of meat due to the presence of essential phytochemicals as phenolic, and flavonoids^2^. Different parts of the plants in general, may be used for nutrition and to protect or treat several diseases^3–5^. In many developing nations, faba beans are a staple food^6^. Faba beans have antioxidant benefits because they contain secondary metabolites^7^. Eating faba beans has been associated with health benefits, such as reducing inflammation, increasing immunity, avoiding fatigue, and avoiding muscle cramps^8^ Faba beans may have anticancer properties and lower blood cholesterol levels^9^. Faba seeds have stiff seed coverings; hence, longer soaking and boiling durations are needed^10^ Soak beans for several hours to remove phytic acid, which will soften the seed coat and reduce cooking time^11^. Cooking techniques increase food safety by destroying microorganisms, improving food digestion, and increasing nutritional bioavailability. However, it can degrade food quality by producing unwanted chemicals and removing some nutrients^12^. Water-soluble phenolic contents were shown to be severely reduced during cooking as a result of their breakdown and flavonoid loss via migration into the cooking water^1^. The cooking process influences the bioactive compounds and antioxidant capacity^13^.Furthermore, using certain chemicals in cooking or soaking processes may influence the nutritional value of faba seeds and could be harmful upon.

According to^14^, a synthetic amino acid called ethylenediaminetetraacetic acid (EDTA) has been used in metal poisoning chelation therapy and other medical applications^15^. Additionally, EDTA is an anti-bacterial and wound-healing agent^16^. Several restaurants have recently shortened the cooking time of faba beans by adding EDTA during the cooking process. The nutritional value of faba seeds may be impacted if EDTA is used while they are cooking^17^. As far as we are aware, this problem is not sufficiently addressed. Therefore, the aim of this study was to evaluate the effects of EDTA on the phytochemical components of faba seeds during the cooking process and extended to look at the adverse effects of cooking media (CM) containing EDTA on mice’s hepato-renal functioning.

## Materials and methods

### Chemicals

Ethylenediamine tetra-acetic acid (EDTA) is an aminopolycarboxylic acid (C_10_H_16_N_2_O_8_, CAS Number: 139-33-3) purchased from Sigma-Aldrich (Sigma-Aldrich, CO. LLC, USA). Faba seeds were purchased from hypermarket (Carrefour, Tanta City, Egypt), andthen the purchased beans were verified by a knowledgeable taxonomist at Tanta University’s Botany Department, Faculty of Science. Catalase (CAT), superoxide dismutase (SOD), reduced glutathione (GSH), malondialdehyde(MDA), alanine aminotransferase (ALT), aspartate aminotransferase (AST), urea and creatinine biochemical kits were purchased from Bio-diagnostic (El-Dokky, Egypt).

### Preparation of faba seeds extracts and cooking media

Sixty grams of faba beans were not treated and the same quantities were boiled with or without EDTA (1 g/L)for 90 min^18^. In each condition, 50 g of air-dried and finely ground faba seeds were soaked in 500 ml of 70% ethanol, thoroughly mixed, and allowed to sit for three days. After filtering, the extracts were allowed to air dry at room temperature.50 g of faba seeds were soaked into 500 ml of distilled water with 1 g EDTA/L, and then boiled for 90 min. Before being used, the CM was filtered and kept at −20 °C. Also, 50 g of faba seeds were combined with 500 ml of distilled water and cooked in the same manner, the CM was utilized as a negative control.

### Determination of phytochemicals content and antioxidant property

The Folin-Ciocalteau reagent method was used to calculate the total phenolic (TP) contents in accordance with^19^. The aluminum chloride colorimetric method was used to determine the total flavonoids (TF) concentration according to^20^. The phosphomolybednum technique was employed to assess the total antioxidant capacity (TAC) of all extracts^21^. DPPH scavenging activity was estimated according to^22^. The following formula was used to determine the inhibition percentage of the DPPH radical’s scavenging activity: Radical scavenging activity % = [(AC –AS)/(AC)] ×100.AC= absorbance of the control (radical solution without sample) and AS= absorbance of the sample (with extract). IC_50_ values were obtained from the concentration–inhibition curve as the concentration corresponding to 50% radical scavenging activity.

### Gas chromatography and mass spectrum analysis

Gas chromatography–mass spectrometry (GC–MS) analysis was performed according to the method previously described by^23^. The Trace GC 1310-ISQ mass spectrometer “GC-MS” (Thermo Scientific, Austin, TX, USA) with a direct capillary column TG-5MS (30 m × 0.25 mm × 0.25 μm film thickness) was used to determine the chemical compositions. The temperature of the column oven was first maintained at 50 °C, then raised by 70 °C per minute to 230 °C and held for two minutes. Finally, it was raised to 300 °C by 30 °C per minute and kept for two minutes. The Auto-sampler (AS1300) in conjunction with GC in split mode was used to automatically inject diluted samples. In full scan mode, mass spectra were obtained at 70 eV ionization voltages across the m/z 45–600 range. By comparing the components’ mass spectra and retention durations to those of the NIST 11 mass spectral database and WILEY 09, the components were identified.

### Mice and experimental design

A total of ninety (20 ± 2 g) male CD-1 mice were purchased from the National Research Center (NRC, Cairo, Egypt). The age of the experimental mice that were used in this study were from 6 to 8 weeks in average. Mice were housed at Tanta University’s Animal Facility, Zoology Department, Faculty of Science. According to the recommended international ethical norms for the care of laboratory animals, experimental design and reporting of data are compliant with the ARRIVE guidelines for in vivo animal research^24^. Mice were acclimatized for one week, then separated into six groups (*n* = 15) as follows. For both sub-acute and sub-chronic administrations, the 1 st and 2nd groups (Gp1 and Gp2) served as negative controls. For 15 days, Gp3 was administered orally along with 200 µl of CM, while, Gp4 were administered with CM/EDTA (10 mg/kg). For 90 days, Gp5 was administered orally with 200 µl of CM and Gp6 was given orally with 200 µl of CM/EDTA (10 mg/kg). All methods were performed in accordance with the relevant guidelines and regulations.

### Preparation of liver homogenate

Immediately after sacrifice, liver tissues were excised, washed with ice-cold normal saline to remove blood residues, blotted dry, and weighed. The tissues were homogenized (10% w/v) in ice-cold phosphate-buffered saline (0.1 M, pH 7.4) using a Teflon-glass homogenizer. The homogenates were centrifuged at 10,000 × g for 15 min at 4 °C, and the resulting supernatants were collected and stored at − 80 °C until used for the determination of oxidative stress parameters.

### Determination of biochemical parameters

Blood samples were collected from the retro-orbital plexus under light anesthesia. The animal is placed in a chamber with a high concentration of anesthetic gas, isoflurane (300 µl of isoflurane in a 500 ml container- chamber for induction (3–5%), and for maintenance (1–3%)^25^. Blood were centrifuged and obtained sera were used for biochemical analysis. Serum ALT and AST activities were determined as described by Thomas^26^, respectively. Kidney functions included urea and creatinine analysis were determined as described by Thomas^26^. Liver tissues were used for oxidative stress level estimation.

Hepatic SOD activity was determined as described by Nishikimi, Rao^27^. CAT activities were measured following the methodology of Aebi^28^. GSH levels were measured according to^29^. MDA was assessed based on the methods that have been previously described by^22^.

### Histopathology investigations

Sections of liver and kidney tissues were promptly preserved in 10% neutral buffered formalin for a full day. The tissue samples were cleaned by xylene, embedded in paraffin, and dehydrated using increasing grades of ethyl alcohol. Hematoxylin and eosin (H&E) was used to stain and mount sections that were 5 µ thick^30^.

### Gene expression analysis

Using β-actin as an internal reference, mRNA expression of nuclear factor Kappa-beta (NFκ-β), tumor growth factor beta-1 (TGF-β1), and cyclooxygenase-1 (COX-1) genes were evaluated in the liver and renal tissues of all groups using real-time PCR with SYBR Green. Maxima SYBR Green/ROX qPCR Master Mix was used to amplify the extracted cDNA according to the manufacturer’s instructions (Thermo Scientific, USA, # K0221). The β-actin gene-specific primers were CGTCACACTTCATGATG (reverse) and ACCCACACTGTGCCCATCTA (forward). For forward and reverse orientations, the corresponding TGFb-1 gene-specific primers were AAGAAGTCACCCGCGTGCTA and TGTGTGATGTCTTT-GGTTTTGTCA. For NFk-β, the particular primers were GGGTCAGAGGCCAATAGAGA (reverse) and CCTAGCTTTCTCTGAACTGCAAA (forward). The forward and reverse Cox-1 primers were CCCAGAGTCATGAGTCGAAGGAG and CAGGCGCATGAGTACTTCTCGG, respectively. These primers were designed using the web-based program using published sequences. BLAST was used to compare the primer sequence to other known sequences.

### Statistical analysis

To evaluate the significant differences between treatment groups, one-way analysis of variance (ANOVA) was employed. SPSS 16.0 software was used to examine percentage data, while the t-test was used to assess data expressed as means ± S.D. For all statistical analyses in this study, a value of *p* < 0.05 was deemed significant.

## Results

### Phytochemical analysis of cooked faba seeds

Hydro-alcoholic extracts of faba seed sections under various treatment conditions were tested for TP, TF, TAC and DPPH scavenging activity. When compared to uncooked faba seed extract (SE), the results demonstrated that compared to uncooked faba seeds (SE), the cooked seeds (SE/C) showed significant reductions (*p* < 0.05) in the TP, TF, TAC and DPPH scavenging activities. Addition of EDTA during the cooking time showed a reduction in TP, TF, TAC and DPPH scavenging activities when compared to the cooked seeds (SE/C) (Table 1).

**Table 1:** Quantitative phytochemical analysis and antioxidant propriety of faba seeds cooked in water or in the presence of EDTA.

IC_50_ of DPPH (mg/ml)	DPPH Scavenging (%)	TAC (mg AAE/g DW)	TF (mg QE/g DW (	TP (mg GAE/g DW)	Conditions
0.91 ± 0.03 ^c^	54.91% ^a^	1.98 ± 0.05 ^a^	17.2 ± 0.5 ^a^	21.5 ± 0.7 ^a^	**SE**
1.2 ± 0.04 ^b^	40.56% ^b^	1.54 ± 0.03 ^b^	12.1 ± 0.45 ^b^	17.5 ± 0.6 ^b^	**SE/C**
1.6 ± 0.05 ^a^	33.02% ^b^	1.05* ± 0.02 ^c^	8.6 ± 0.39 ^c^	11.2 ± 0.4 ^c^	**SE/C/EDTA**

The values represented mean ± SD; SE: Seeds extract (uncooked); SE/C: Seeds extract (cooked); SE/C/EDTA: Seeds extract cooked in presence of EDTA; TP: Total phenolic, TF: Total flavonoid; TAC: Total antioxidant capacity; DPPH: Diphenyl-1-picrylhydrazyl; IC_50_: Inhibitory concentration of 50%. GAE: Gallic acid equivalent; QE: Quercetine equivalent; AAE: Ascorbic acid equivalent; DW: Dry weight. Means that do not share a letter are significant difference. P value < 0.05 was statistically significant.

### GC-MS analysis of cooked faba seeds extract

While the ethyl α-D-glucopyranoside molecule was not detected in either SE/C or SE/C/EDTA, the GC-MS analysis revealed that it was present in faba seed extracts (SE) and had a peak area (P.A. = 40%) at the retention time (RT: 9.23 min). Furanacetic acid, 4-hexyl-2,5-dihydro-2,5-dioxo and ethyl-1,3-dioxane-5-methanol, tetra-butyl di-methylsilyl ether were reduced in SE/EDTA. Adding EDTA (SE/C/EDTA) led to disappear of these compounds. Only SE/C/EDTA contains di-isooctyl phthalate (DIOP) (P.A. = 63.8%) (Table 2).

**Table 2:** GC-MS analysis of faba seeds extract cooked in absence or presence of EDTA.

SE/C/ EDTA	SE/C	SE	M.Wt	M.F	Name	*R*.T (min)
Peak area (*P*.A) %						
ND	1.0	3.67	240	C_12_H_16_O_5_	3-Furanacetic acid, 4-hexyl-2,5-dihydro-2,5-dioxo	5.96
ND	0.7	25.56	260	C_13_H_28_O_3_Si	5-Ethyl-1,3-dioxane-5-methanol, tetra-butyldimethylsilyl ether	7.86
ND	ND	40.80	208	C_8_H_16_O_6_	Ethyl α-D-glucopyranoside	9.23
0.49	4.9	0.76	270	C_17_H_34_O_2_	Pentadecanoic acid, 14-methyl-methyl ester	10.8
1.14	0.3	0.71	270	C_17_H_34_O_2_	Hexadecanoic acid, methyl ester	11.5
1.61	19	0.54	296	C_19_H_36_O_2_	Methyl octadeca-9,12-dienoate	127
1.47	0.6	1.19	310	C_20_H_38_O_2_	(E)−9-Octadecenoic acid ethyl ester	13.4
0.75	2.7	0.90	356	C_21_H_40_O_4_	9-Octadecenoic acid (Z)-, 2,3-dihydroxypropyl ester	14.9
8.12	9.2	3.92	143	C_7_H_13_NO_2_	2-Propenoic acid, 2-(dimethylamino)ethyl ester	16.1
63.83	ND	ND	390	C_24_H_38_O_4_	Diisooctyl phthalate	16.9
4.41	3.7	ND	356	C_21_H_40_O_4_	9-Octadecenoic acid (Z)-, 2-hydroxy-1-(hydroxymethyl)ethyl ester	18.4
2.17	3.6	ND	416	C_28_H_48_O_2_	α-Tocopherol	23.5
5.96	11	1.86	414	C_29_H_50_O	α-Sitosterol	27.9

R.T: Retention time; M.F: Molecular formula; M.Wt: Molecular weight; SE: Seeds extract (uncooked); SE/C: Seeds extract (cooked); SE/C/EDTA: Seeds extract cooked in presence of EDTA.

### Treatment of mice with CM/EDTA did not influence body weight

**Fig. 1:**
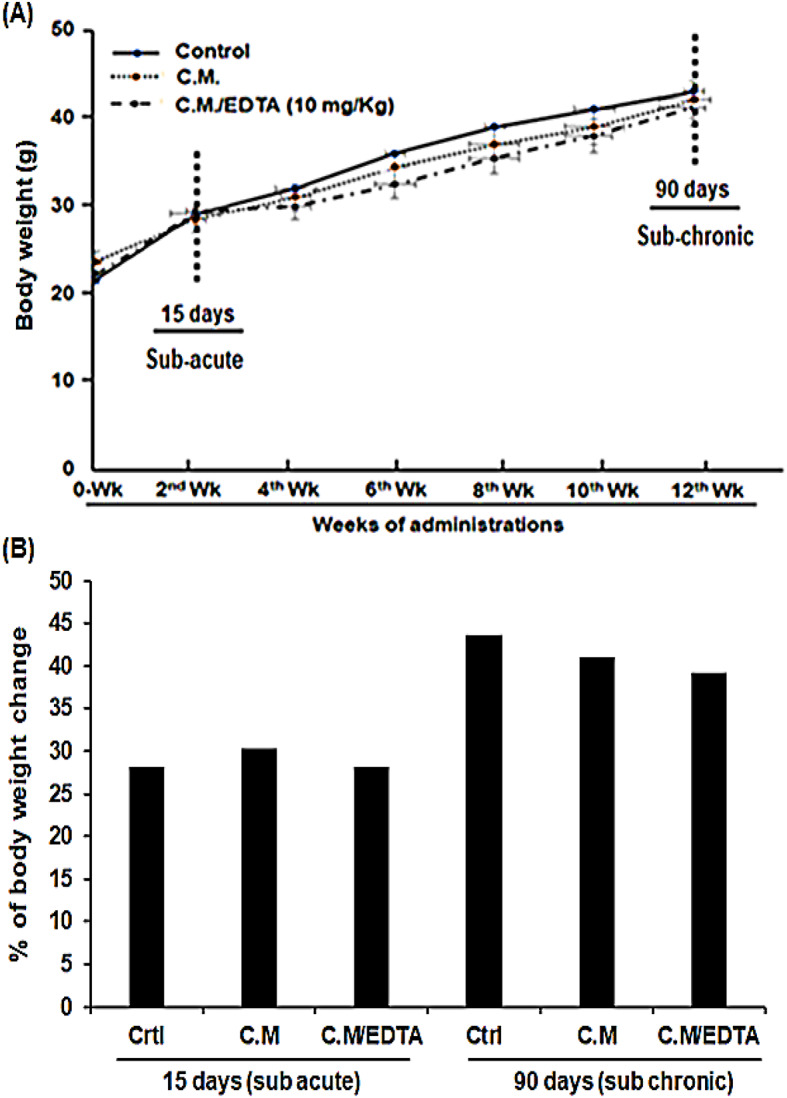
Kinetics of body weight changes post sub-acute and sub-chronic administrations of CM. alone or CM. containing EDTA for 15 and 90 days (**A**). The percentages of body weight changes post sub-acute and sub-chronic administrations of C.M. alone or C.M. containing EDTA (**B**).

When compared to the normal control groups (Gp1 and Gp2), the percentage of body weight changes (% b.wt.) did not changed after administration of CM for 15 or 90 days. As compared to the Gp1 and Gp2, the % b.wt was decreased as a result of sub-acute or sub-chronic administration of CM/EDTA (Fig. 1A). After 15 days, the % b.wt changes in Gp1, Gp3, and Gp4 were 28.1, 30.3, and 28.2%, respectively. However, post 90 days, the % b.wt. in Gp2, Gp5, and Gp6 were43.6%, 41.1% and 40.2%, respectively(Fig. 1B).

### Administration of CM/EDTA altered liver and kidney functions

As compared to Gp1, the liver transaminases (ALT, AST) activities, urea, and creatinine levels did not alter significantly after 15 days of administration with CM alone (Gp3) or CM/EDTA (Gp4). When compared toGp2, these biochemical parameters were unchanged after 90 days of administration with CM alone (Gp5).While, the sub-chronic administration of CM/EDTA (10 mg/kg) (Gp6) increased these values in sera (Table 3).

**Table 3:** Serum ALT, AST activities, urea, and creatinine levels post 15 and 90 days of administration.

Creatinine (mg/dl)	Urea (mg/dl)	AST (U/L)	ALT (U/L)	Groups
15-days post administrations (Sub-acute)				
0.42 ± 0.06	42.5 ± 0.6	210.8 ± 6.5	52.3 ± 1.7	Ctrl
0.45 ± 0.08	46.3 ± 0.9	233.5 ± 5.3	52.3 ± 1.0	CM alone
0.41 ± 0.05	48.7 ± 0.7	242.5 ± 6.3	42.3 ± 1.3	CM/EDTA
90-days post administrations (Sub-chronic)				
0.35 ± 0.06	38.89 ± 0.9	179.6 ± 2.1	49 ± 0.9	Ctrl
0.49 ± 0.02	41.24 ± 0.8	185.5 ± 3.2	62 ± 1.1	CM alone
0.87 ± 0.03*	48.35 ± 1*	197.5 ± 2.8	88 ± 0.5*	CM/EDTA

Ctrl: Control, CM: Cooking media, ALT: Alanine transaminase, AST: Aspartate transaminase. * P value < 0.05 was statistically significant.

### Sub-chronic administration of CM/EDTA altered oxidants/antioxidants hemostasis

**Fig. 2:**
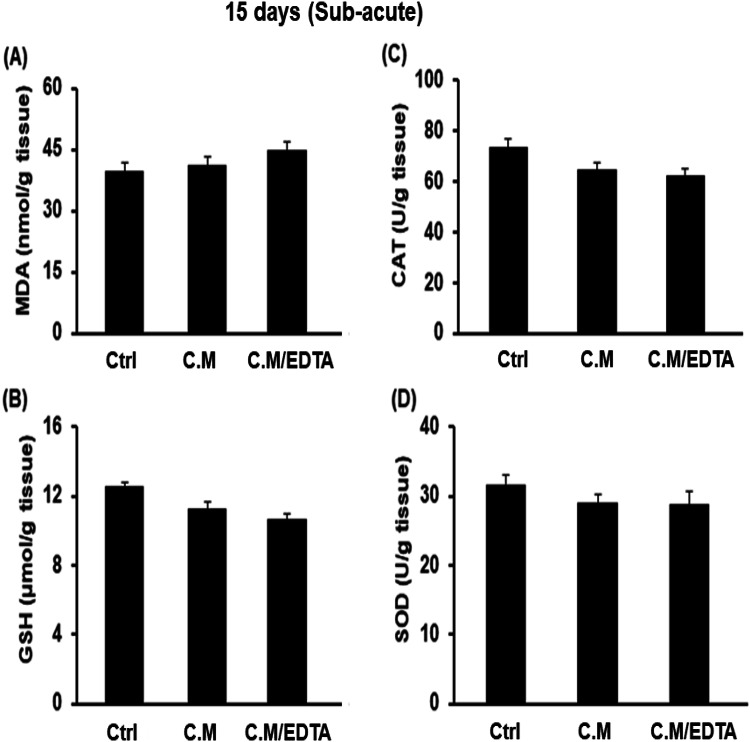
Hepatic oxidative stress biomarkers post 15 days of administration with CM. alone or CM. containing EDTA. MDA levels (**A**), GSH levels (**B**), CAT activity (**C**), and SOD activity (**D**) in all groups.

**Fig. 3:**
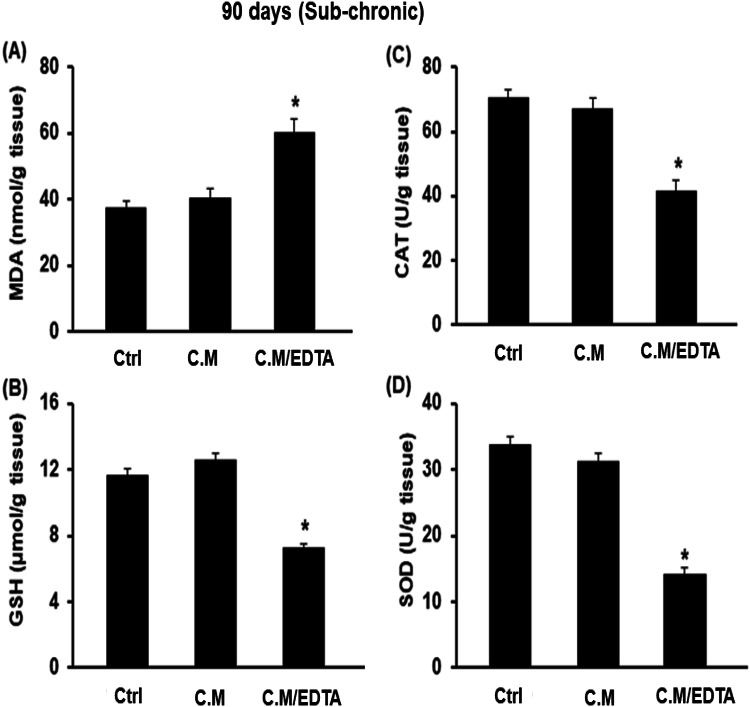
Hepatic oxidative stress biomarkers post 90 days. (**A**) MDA levels, (**B**) GSH content, (**C**) CAT activity, and (**D**) SOD activity in all groups.

In the liver tissues, oxidant/antioxidant biomarkers (MDA, GSH, CAT, and SOD) were evaluated 15 and 90 days after dosing. Sub-acute administrations of CM or with EDTA did not change these biomarkers, according to the results (Fig. 2). However, when compared to Gp2, sub-chronic administration of CM/EDTA resulted in a significant (*p* < 0.05) increase in MDA levels and a decrease in GSH content, SOD, and CAT activities (Fig. 3).

### Administration of CM/EDTA for 90 days altered the architectures of liver and kidney tissues this section should be before molecular analysis

**Fig. 4:**
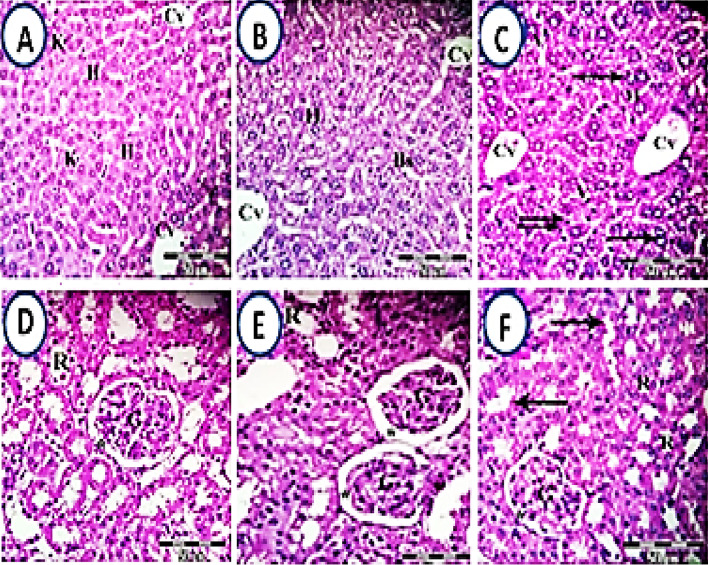
(**A-F**): Photomicrographs of liver and kidney sections of different groups administered for 15 days (**H** & **E**). Liver sections of control group (**A**) and CM administrated mice (**B**) showing normal hepatocytes (H), central vein (CV), Kupffer cells (K), and blood sinusoids (Bs). Liver sections of mice administered with CM/EDTA (**C**) showing few cellular infiltrations (arrow). Kidney sections of control group (**D**) and CM administrated mice (**E**) showing normal renal architecture (R) with regular glomeruli (G) and Bowman´s capsules. Kidney sections of mice administered with CM/EDTA (**F**) showing few dilated and damaged renal tubules (arrows).

**Fig. 5:**
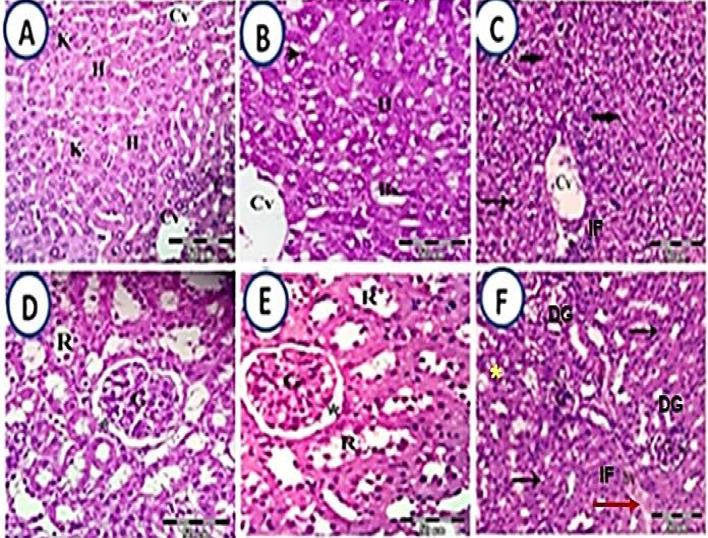
(**A-F**): Photomicrographs of liver and kidney sections of different groups administered for 90 days (**H** & **E**). Liver sections of control group (**A**) and CM administrated mice (**B**) showing normal hepatocytes (H), central vein (CV), blood sinusoids (Bs), and Kupffer cells (K). Liver sections of mice administered with CM/EDTA (**C**) showing hepatic tissue alterations, degeneration, pyknotic hepatocytes (arrows), cytoplasmic vacuolation, karyolitic nuclei and congested central vein (CV) with cellular infiltration (IF). Kidney sections of control group (**D**) and CM administrated mice (**E**) showing normal renal architecture (R) with normal glomeruli (G). Kidney sections of mice administered with CM/EDTA (**F**) showing renal histopathological alterations, degenerated renal tubules (DR), and atrophic renal glomeruli (*). Several pyknotic cells (black arrows) and inflammatory cells (IF). In addition, congested blood vessels (red arrow).

The liver sections of mice given CM for 15 or 90 days displayed normal central veins (Cv), normal hepatic strands (H), some hepatocytes that were degenerated and vacuolated (V), while others were binucleated (arrows), and regular blood sinusoids with distinct Kupffercells (Figs. 4A and B and 5A and B). Mice given CM/EDTA for 15 days (Gp4) showed a well-organized hepatic architecture and central vein (Cv), with a few cellular infiltrations (arrow) and Kupffer cells (K) visible in the liver section (Fig. 4C). Mice given CM-containing EDTA for 90 days (Gp6), showed certain histological changes in their liver sections resulting in cytoplasmic vacuolation, cellular infiltration, cellular disarray, degeneration, and pyknosis of many hepatocytes, particularly in the centrilobular zone (Fig. 5C).

Control mice’s kidney sections displayed normal renal architecture, including Bowman’s capsules and typical glomeruli (Fig. 4D). In contrast, the renal sections of CM (Gp3) or CM/EDTA (Gp4) for 15 days showed normal renal tissue structure, normal glomeruli with regular Bowman’s space, and a majority of normal renal tubules (R) with a small number of dilated and damaged ones (arrows) (Fig. 4E and F). Figure 5D and E show kidney slices from mice given CM and control mice that have normal-like renal tissue architecture. On the other hand, kidney sections from mice given CM/EDTA for 90 days showed damaged Bowman’s capsules and disordered glomeruli (G). Histopathological changes in the renal tubules included atrophy and destruction of the lining epithelia (R); other findings included inflammatory cell infiltrations and many pyknotic cells with darkly stained nuclei. Congested blood arteries were also observed (Fig. 5F).

### Molecular analysis of pro-inflammatory cytokines using RT-PCR

In comparison to their controls (Gp1 and Gp2), administration of CM/EDTA for 15 days did not significant changes (*p* > 0.05) the mRNA expression levels of the pro-inflammatory genes (TGF-β1, NFk-β, and COX-1) in the liver and kidney tissues (Table 4). In contrast, CM containing EDTAadministration for 90 days showed significant increase in the expression levels of these genes in their liver and renal tissues (Table 4).

**Table 4:** Fold changes of the mRNA expression of TGFβ−1, NFκ-β, and COX-1 genes in liver and kidney tissues of the different groups.

Groups, 15-days post administrations (Sub-acute)			Tissue/Genes	
CM/EDTA	CM alone	Ctrl		
1.21 ± 0.27	1.13 ± 0.17	1.05 ± 0.14	TGFβ−1	Liver
1.32 ± 0.34	1.27 ± 0.15	1.04 ± 0.15	NFκ-β	
1.14 ± 0.15	1.08 ± 0.16	1.03 ± 0.15	COX-1	
1.11 **±** 0.18	1.03 ± 0.12	1.06 ± 0.16	TGFβ−1	Kidney
1.07 ± 0.31	1.03 ± 0.11	1.01 ± 0.14	NFκ-β	
1.15 ± 0.28	1.06 ± 0.13	1.04 ± 0.16	COX-1	

Groups, 90-days post administrations (Sub-chronic)			Tissue/Genes	
CM/EDTA	CM alone	Ctrl		
4.13 ± 0.27**	1.313 ± 0.19*	1.05 ± 0.14	TGFβ−1	Liver
4.71 ± 0.34***	1.27 ± 0.13**	1.04 ± 0.15	NFκ-β	
3.08 ± 0.15**	1.28 ± 0.14*	1.03 ± 0.15	COX-1	
2.61 ± 0.18*	1.23 ± 0.12*	1.06 ± 0.16	TGFβ−1	Kidney
3.17 ± 0.31**	1.13 ± 0.15*	1.01 ± 0.14	NFκ-β	
2.55 ± 0.28*	1.21 ± 0.14*	1.04 ± 0.16	COX-1	

Ctrl: Control, CM: Cooking media, * P value < 0.05 was statistically significant.

## Discussion

This study found that adding EDTA to faba beans during the boiling process changed their phytochemical compositions, and that administering CM/EDTA altered the biochemical and histological levels in mice’s liver and kidney tissues. A recent study found that adding EDTA to water during heating lowers the macro- and micronutrients in faba seeds^17^. This study investigated how the addition of EDTA during the cooking process altered the phytochemical composition of faba seed. According to this study, the high quantities of flavonoids and phenolic compounds in faba seeds, indicating their potential as natural antioxidants^31^ In contrast, EDTA reduced these secondary metabolites. This finding was consistent with another study, which demonstrated that cooking altered the phytochemical content and antioxidant capacity of beans and vegetables^32^. The boiling procedure itself lowered the amount of TP, TF, TAC, and DPPH scavenging materials. These findings were consistent with earlier research, which showed that cooking faba bean seeds in water reduced their phytochemical concentration^33^. When EDTA was added, the previous phytochemical readings fell much lower. It has been demonstrated that cooking causes a decrease in phytochemical content due to the leaching of these compounds into the cooking medium^12^. EDTA-chelating activity may be the cause of the decrease in the prior values following the addition of EDTA during the cooking phase.

Another element to consider is the development of novel chemical compounds^17^. EDTA-chelating activity and the formation of novel chemical compounds may be responsible for the decrease in prior metrics observed with the addition of EDTA during the cooking process^17^. Furthermore, the leaching of these compounds into the cooking water may be responsible for the decreased or reduced phytochemical components of cooked faba seeds in the presence of EDTA.Uncooked faba seeds include phytochemicals such as tetra-butyldimethylsilyl ether, α-sitosterol, fatty acids, ethyl α-D-glucopyranoside, 5-Ethyl-1,3-dioxane-5-methanol, 4-hexyl-2,5-dihydro-2,5-dioxo, 3-furanacetic acid, and others. This result was consistent with earlier research that used GC/MS screening to detect comparable secondary metabolites in faba seeds^34^. According to Van Noten, Degroote^35^, these health-promoting substances are regarded as functional health enhancers. According to GC-MS research, heating faba seeds lowered the amount of various phytochemical compounds; interestingly, DIOP, 9,10-Secocholesta-5,7,10(19), and other unique phytochemical compounds were generated when EDTA was applied during faba seed roasting. These chemicals may have formed as a result of an interaction between EDTA and other phytochemical components during the cooking process of faba beans. These chemicals in faba seeds or cooking media could be harmful to human health. The presence of DIOP may be significant due to its documented biological activities and potential toxicological consequences, which could add to the extract’s overall pharmacological or toxicological profile^36^-^38^. DIOP has the highest peak area among these new compounds. 63.83% demonstrated mutagenic, neurotoxic, and toxicity^39^.

The antioxidant potential of faba seeds may vary depending on whether certain phytochemical components are reduced, enhanced, or decreased after heating^13^. Prolonged oral EDTA administration can be dangerous, depending on the dosage and manner of delivery^40^. Cooking with chemicals decreases the nutritional value of faba seeds and may have negative health consequences^18^. The administration of CM alone for either 15 days (Sub-acute) or 90 days (Sub-chronic) had no significant effect on body weight alterations in the current investigation when compared to their control group. The hematological parameters were unaltered after 15 days of EDTA-containing C.M., although the differential leucocytes changed after 90 days. This observation may be due to the harmful activity of EDTA metabolites on the spleen or bone marrow. EDTA may alter hematological parameters following subchronic dosing, according to previous study^18^. Following CM dosing, there was no change in the biomarkers for ALT, AST, urea, and creatinine. After 15 days of either CM or CM with EDTA, the biomarkers for ALT, AST, urea, and creatinine did not change; however, after 90 days of CM with EDTA, these parameters were increased.

According to these results, the administration of CM/EDTA changed the physiological and biochemical characteristics of the liver and kidney tissues. This could be because boiling EDTA with beans produces some harmful chemicals. These results concurred with those of^18^, who found that administering CM/EDTA raised biomarkers for the liver and kidneys. Multiple intravenous EDTA chelation therapy with or without vitamin C have been shown in previous studies to reduce oxidative stress and lipid peroxidation^41^.

After 15 days of treatment with CM alone or CM/EDTA, the levels of oxidants/antioxidants biomarkers in the current study remained stable. Because of the shift in oxidant/antioxidant balance, these findings revealed that while sub-acute CM treatment with EDTA was not dangerous, post-90-day (sub-chronic) administration was. When CM is delivered, the antioxidant balance is disrupted. Mice’s exposure to EDTA could be due to the negative effects of newly formed compounds during cooking, which occurred from EDTA’s interaction with faba seed phytochemicals on enzyme activity. The study found that providing CM/EDTA for 15 days did not affect the expression of pro-inflammatory genes (NFk-β, TGF-β1, and COX-1) in the liver and kidney tissues. However, administering it for 90 days enhanced their expression. This could be due to long-term negative effects of treatment. The use of EDTA caused histopathological alterations in experimental mice^40^.The current study found that, consistent with the hematological and biochemical results, administering CM/EDTA for 15 days did not change the architecture of the liver or kidney; however, after 90 days, administering CM containing EDTA (10 mg/Kg) caused histopathological changes in the liver and kidney tissues. According to Ramadan, Sakr^40^, giving mice an EDTA-contaminated diet caused interstitial cell degeneration and lowered fertility. When EDTA was added to roasted faba seeds, the phytochemical composition changed and antioxidant capability dropped. Furthermore, when EDTA was added to faba beans during the cooking process, new chemical components were generated, which could have a direct impact on human health. It is recommended that this study look at the potential health impacts of the new compounds produced when EDTA is added to faba beans during cooking.

## Conclusion

This study reported that adding EDTA to faba beans during the cooking process altered their phytochemical content and reduced antioxidant potency. Sub-acute administration of EDTA-containing CM at a dose of 10 mg/kg had no significant effect on the biochemical and histological assessments of experimental mice’s liver and kidney tissues. In contrast, sub-chronic treatment altered the structures and activities of the kidney and liver tissues. In this investigation, we were unable to separate newly generated chemical compounds such as DIOP from the CM and investigate their toxicity in animal models. Furthermore, this study did not address the chronic administration of CM/EDTA and its impact on the hepatic and renal structures and functionality.

## Data Availability

The datasets generated and/or analyzed during the current study are available upon reasonable request.
